# Improved perioperative outcomes and reduced inflammatory stress response in malignant robot-assisted colorectal resections: a retrospective cohort study of 298 patients

**DOI:** 10.1186/s12957-021-02263-w

**Published:** 2021-05-22

**Authors:** Pedja Cuk, Randi Maria Simonsen, Mirjana Komljen, Michael Festersen Nielsen, Per Helligsø, Andreas Kristian Pedersen, Christian Backer Mogensen, Mark Bremholm Ellebæk

**Affiliations:** 1 Department of Surgery, Hospital of Southern Jutland, Aabenraa, Denmark; 2 Department of Regional Health Research, Hospital of Southern Jutland, University of Southern Denmark, Odense, Denmark; 3 OPEN, Odense Patient data Explorative Network, Odense University Hospital, Odense, Denmark; 4 Research Unit for Surgery, Surgical Department, Odense University Hospital, Odense, Denmark

**Keywords:** Colorectal cancer, Robot-assisted colorectal surgery, Laparoscopic colorectal surgery, Postoperative inflammatory stress response, Surgical oncology, Minimally invasive surgery

## Abstract

**Background:**

Robot-assisted surgery is increasingly implemented for the resection of colorectal cancer, although the scientific evidence for adopting this technique is still limited. This study’s main objective was to compare short-term complication rates, oncological outcomes, and the inflammatory stress response after colorectal resection for cancer performed laparoscopic or robot-assisted.

**Methods:**

We conducted a retrospective cohort study comparing the robot-assisted approach to laparoscopic surgery for elective malignant colorectal neoplasm. Certified colorectal and da Vinci ® robotic surgeons performed resections at a Danish tertiary colorectal high volume center from May 2017 to March 2019. We analyzed the two surgical groups using uni- and multivariate regression analyses to detect differences in intra- and postoperative clinical outcomes and the inflammatory stress response.

**Results:**

Two hundred and ninety-eight patients were enrolled in the study. Significant differences favoring robot-assisted surgery was demonstrated for; length of hospital stay (4 days, interquartile range (4, 5) versus 5 days, interquartile range (4–7), p < 0.001), and intraoperative blood loss (50 mL, interquartile range (20–100) versus 100 mL, interquartile range (50–150), p < 0.001) compared to laparoscopic surgery. The inflammatory stress response was significantly higher after laparoscopic compared to robot-assisted surgery reflected by an increase in C-reactive protein concentration (exponentiated coefficient = 1.23, 95% confidence interval (1.06–1.46), p = 0.008). No differences between the two groups were found concerning mortality, microradical resection rate, conversion to open surgery, and surgical or medical short-term complication rates.

**Conclusion:**

Robot-assisted surgery is feasible and can be safely implemented for colorectal resections. The robot-assisted approach, when compared to laparoscopic surgery, was associated with improved intra- and postoperative outcomes. Extensive prospective studies are needed to determine the short- and long-term outcomes of robotic surgery for colorectal cancer.

## Introduction

Colorectal cancer is a common malignant disease with a global annual incidence of 1.8 million (2017) and an incidence rate of 23.2/100,000 inhabitants [1]. The treatment approach is primarily surgical, and if possible, a minimally invasive approach should be preferred. Laparoscopic colorectal surgery (LCS) has existed for more than 20 years. The many benefits of LCS compared to open surgery include less postoperative pain, fewer wound complication rates, and improved cosmetic outcomes [2]. The disadvantages of LCS include a prolonged learning curve, suboptimal ergonomics, and suboptimal visual exposure of the surgical field due, in part, to tremor caused by the assistant [3–5]. The oncological results of LCS and open colorectal surgery are identical, and the 3 to 5-year survival and recurrence rates of the two surgical methods are comparable [2].

2Within the last 15 years, robot-assisted colorectal surgery (RCS) has increasingly been used to treat colorectal cancer. Weber et al. was the first to describe this surgical method in 2002 [6]. RCS is associated with longer operating times and higher total costs when compared to laparoscopic surgery. However, robot-assisted surgery has additional benefits, including a reduced risk of conversion to open surgery, improved postoperative morbidity, and reduced intraoperative bleeding, comparable oncologic rates, and a faster establishment of bowel function [7–11]. Furthermore, robot-assisted surgery offers a stable and better visual exposure, and instruments with a higher degree of flexibility to enhance surgical dissection quality [7, 12]. It can be challenging to determine the differences between intra- and postoperative outcomes as both surgical methods are minimally invasive. The incidence of lower morbidity rates in RCS [13, 14] compared to LCS surgery may be associated with a lower inflammatory stress response due to less tissue trauma and better hemostasis [15]. Therefore, we hypothesized that RCS induces a lower surgical trauma with improved surgical morbidity than LCS.

This study’s main objective was to compare short-term complication rates, oncological outcomes, and inflammatory stress response after resection for cancer performed with robot-assisted or laparoscopic colorectal surgery.

## Methods

### Study design

The study was conducted according to The Strengthening the Reporting of Observational Studies in Epidemiology (STROBE guidelines) [16]. It was performed as a retrospective cohort study at the Surgical Department, Hospital of Southern Jutland, Denmark — a tertiary care hospital performing robot-assisted and laparoscopic colorectal surgery. We minimized bias by only including procedures performed by certified surgeons in colorectal cancer and robot-assisted surgery (da Vinci®, Intuitive Surgical, Inc., Sunnyvale, CA, USA). All surgeons underwent formalized robotic simulator training, and were subsequently supervised by certified colorectal surgeons with experience in robot-assisted colorectal surgery during the initial surgical procedures. Data collection was obtained by review of medical records from May 2017 to March 2019. The study material has been consistently analyzed since the implementation of robot-assisted surgery in our institution. Only patients undergoing intended curative elective colorectal resections were included in the study. Established search criteria were endoscopic and histopathological confirmed adenocarcinoma of the colon (cecum, right colon, transverse colon, left colon, sigmoid colon) and rectum. We excluded patients who underwent palliative surgery. These were patients with unresectable cancer, if permanent diverting stoma or bypass surgery was needed, and if they presented with non-curable metastatic disease. Patients were also excluded if an emergent surgical intervention was required such as cases of pneumoperitoneum, mechanical bowel obstruction, ischemia, or if abscess formation was suspected. Patients presenting with benign conditions (diverticular disease, inflammatory bowel disease, and functional bowel disorders) were also excluded.

### Outcome measurements and data collection

Data were registered in an electronic database, Research Electronic Data Capture (REDCap®), hosted by the Open Patient data Explorative Network (OPEN), Odense University Hospital, and Department of Clinical Research [17]. We collected information regarding demographics (age, gender, body mass index (BMI), and the American Society of Anesthesiologists (ASA) physical status classification system). Intra- and postoperative data included the following parameters: conversion to open surgery, estimated intraoperative blood loss, operative time, length of hospital stay, time to first flatus and stool, and surgical and medical complication rates. Postoperative surgical and medical complication rates occurring within 90 days postoperatively were defined as severe if graded ≥ 3 points according to Clavien Dindo classification [18]. Anastomotic leakage was graded in severity according to Rahbari from A (conservative treatment), B (active re-intervention without laparoscopy or laparotomy), and C (re-intervention with laparoscopy or laparotomy) [19]. The leakage between intestinal walls of anastomosis ends were detected by either (1) pneumoperitoneum on computer tomography (CT) or (2) dehiscence between intestinal ends identified by either re-laparoscopy, re-laparotomy, or endoscopy. Pathological data included the TNM classification of malignant tumors (TNM), number of harvested lymph nodes, rate of microradical resection, and preoperative neoadjuvant chemotherapy or combined chemo-/radiotherapy. To evaluate the inflammatory stress response, CRP and leukocyte counts were registered from the first postoperative day until discharge. Patients were consistently discharged if intestinal function (stools) was established, they could consume solid food, and had satisfactory paraclinical parameters (infection control, electrolyte- and hydration status). In terms of pain control, patients were discharged when only oral analgesics were necessary for pain management.

### Surgical procedure

Patients were allocated to either RCS or LCS depending on the first available scheduled surgical time. Before surgery, patients underwent a multidisciplinary cancer conference. All patients received preoperative antibiotic prophylaxis (Piperacillin/Tazobactam 3 + 0.5 g and Metronidazole 1.5 g), anti-thrombotic (Dalteparin 5000 IE), compression stockings, urinary catheter, and a nasogastric tube. Surgical procedures were performed either laparoscopically or totally robot-assisted. A da Vinci® robot, Xi model (Intuitive Surgical, Inc., Sunnyvale, CA, USA), was used in the RCS procedures. Electric scissors were used for lateral dissection in both surgical groups. In medial dissection, a Ligasure ® device was used in LCS and a Vessel Sealer device ® in RCS. Two-dimensional or three-dimensional (3D) laparoscopy was used in LCS procedures depending on the surgeon’s choice. Complete mesocolic excision (CME) was not applied, but a minimum D2 resection was performed in all colonic resections. Low or abdominoperineal anterior resections were based on total or partial mesorectal excision principles. The following trocars were applied in RCS procedures: a 12-mm umbilical trocar for camera guidance, three 8-mm trocars for surgical instruments, and one 8-mm trocar for the surgical assistant. Traditionally in LCS, four or five 5- and 12-mm trocars were applied. A transverse muscle splitting incision was performed for right-sided tumors, the specimen was extracted, and an extra-corporeal isoperistaltic single-layer, end-to-end, hand-sewn anastomosis was performed. A horizontal incision in the left iliacal fossa was used for specimen extraction in left-sided and rectal tumors, and an end-to-end stapled anastomosis was performed. In low anterior resection procedures, a protective loop ileostomy was performed, dependent on patients’ comorbidity or in case of a low colorectal anastomosis (< 5 cm).

### Statistics

Descriptive statistics were performed for each variable. Categorical variables were presented with frequencies and percentage and compared using Fisher’s exact or Pearson chi-square test depending on Cochrane’s rule [20]. Non-categorical variables were presented with median and interquartile range and compared using the Wilcoxon rank sum test. Logistic regression was used to examine the complication rates between the two surgical techniques. Depending on the outcome’s distribution, Poisson regression or a negative binomial regression was used to examine how the different surgical techniques influenced the length of hospital stay, time to first flatus, stool, and harvested lymph nodes. Log transformation with a mixed effect model estimated CRP and leukocyte differences between the two operation methods across all days. All generalized linear models were adjusted for T-stage, ASA-score, neoadjuvant chemotherapy, BMI, age, type of resection (colonic or rectal), and temporary diverting ileostomy formation. Models followed the one in ten rule for fixed effects and the one in twenty rule for random effects. The models’ fit was completed using quantile-quantile plots of the deviance residuals and residuals for each time point for the generalized linear model and mixed effect models, respectively. A p-value of less than 0.05 was seen as statistically significant, and no correction for multiple testing was utilized. The STATA 16 software (StataCorp, 2019, Stata Statistical Software: Release 16, College Station, TX, StataCorp, LLC) was used to perform the statistical analyses.

## Results

**Fig. 1:**
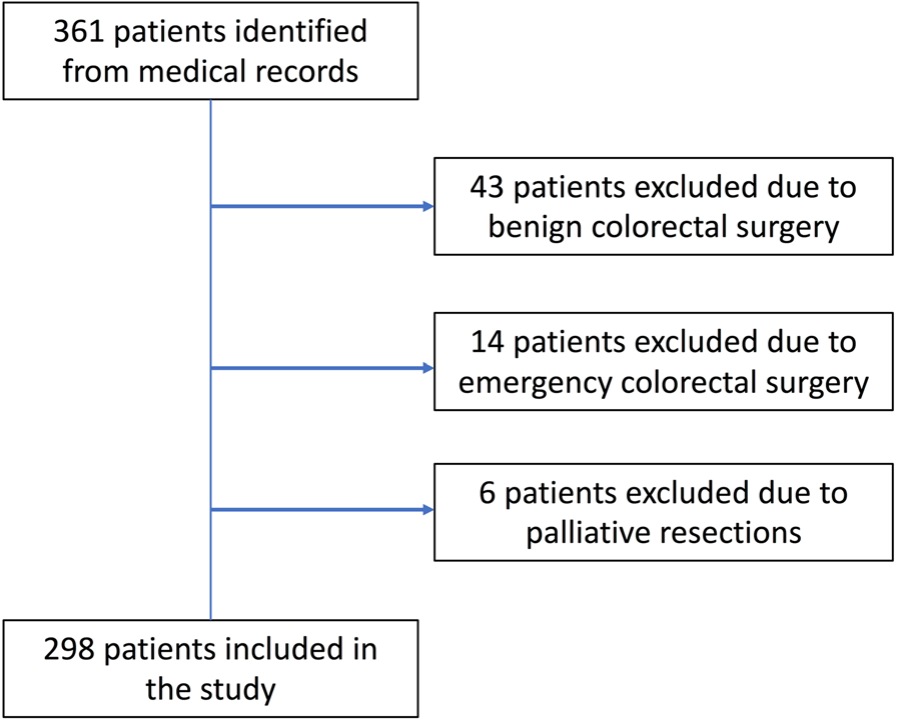
Flowchart of study population selection

Three-hundred and sixty-one patients operated for colorectal cancer were identified, and of these, 298 patients (RCS, n = 143 (48%); LCS, n = 155 (52%)) fulfilled the inclusion criteria (Fig. 1). All baseline characteristics were similar across the two groups except for neoadjuvant chemotherapy (RCS group (7.0%) versus LCS group (16.8%), p = 0.010) and combined neoadjuvant chemo-/radiotherapy (RCS group (4.2%) versus LCS group (13.5%), p = 0.005). The amount of patients who underwent a protective diverting ileostomy was significantly higher in the LCS group compared to the RCS group (40.9% versus 25%, p = 0.002) (Table 1).

**Table 1:** Demographic characteristics. *RCS* robot-assisted colorectal surgery, *LCS* laparoscopic colorectal surgery

Patient characteristics	Level	RCS (n = 143)	LCS (n = 155)	p-value
Sex, n (%)	Male	69 (48.3%)	86 (55.5%)	0.212^a^
	Female	74 (51.7%)	69 (44.5%)	
Age, median (IQR)		70 (61–70)	71 (64–78)	0.116^b^
BMI, median (IQR)		27 (24–30)	26 (24–28)	0.116^b^
ASA grade, n (%)	1	12 (8.4%)	12 (7.7%)	0.124^a^
	2	70 (49.0%)	62 (40.0%)	
	3	59 (41.3%)	72 (46.5%)	
	4	2 (1.4%)	9 (5.8%)	
Neoadjuvant chemotherapy, n (%)		10 (7.0%)	26 (16.8%)	0.010^a^
Temporary diverting ileostomy, n (%)		8 (25%)	27 (40.9%)	0.002^a,c^
Neoadjuvant combined chemo/radiotherapy, n (%)		6 (4.2%)	21 (13.5%)	0.005^a^

^a^*χ*^2^-test

^b^Wilcoxon rank sum test

^c^In case of rectal resection

### Intraoperative outcomes

There was a statistically significant difference in the distribution of colonic and rectal resections between the two surgical methods (RCS group (77.6%) versus LCS group (57.4%), p = 0.015) (Table 2). Median intraoperative blood loss was 50 mL (20–100) in the RCS group versus 100 mL (50–150 mL) in the LCS group, p < 0.001 (Table 2). There were significantly more patients who underwent an abdominoperineal resection in the LCS group compared to the RCS group (33.3% versus 25%, p = 0.014).

**Table 2:** Comparison of intraoperative clinical characteristics of included patients operated for malignant colorectal neoplasm. *RCS* robot-assisted colorectal surgery, *LCS* laparoscopic colorectal surgery

Operative and intraoperative details	Level	RCS (n = 143)	LCS (n = 155)	p-value
Colonic resections, n (%)		111 (77.6%)	89 (57.4%)	0.015^a^
Rectal resections, n (%)		32 (22.4%)	66 (42.6%)	
Conversion rate, n (%)		2 (1.4%)	6 (3.9%)	0.187^a^
Operative blood loss, median (IQR), mL		50 (20–100)	100 (50–150)	< 0.001^b^
Operative time, median (IQR), min		248 (209–310)	277 (196–360)	0.190^b^

^a^*χ*^2^-test

^b^Wilcoxon rank sum test

### Postoperative outcomes and complication rates

**Fig. 2:**
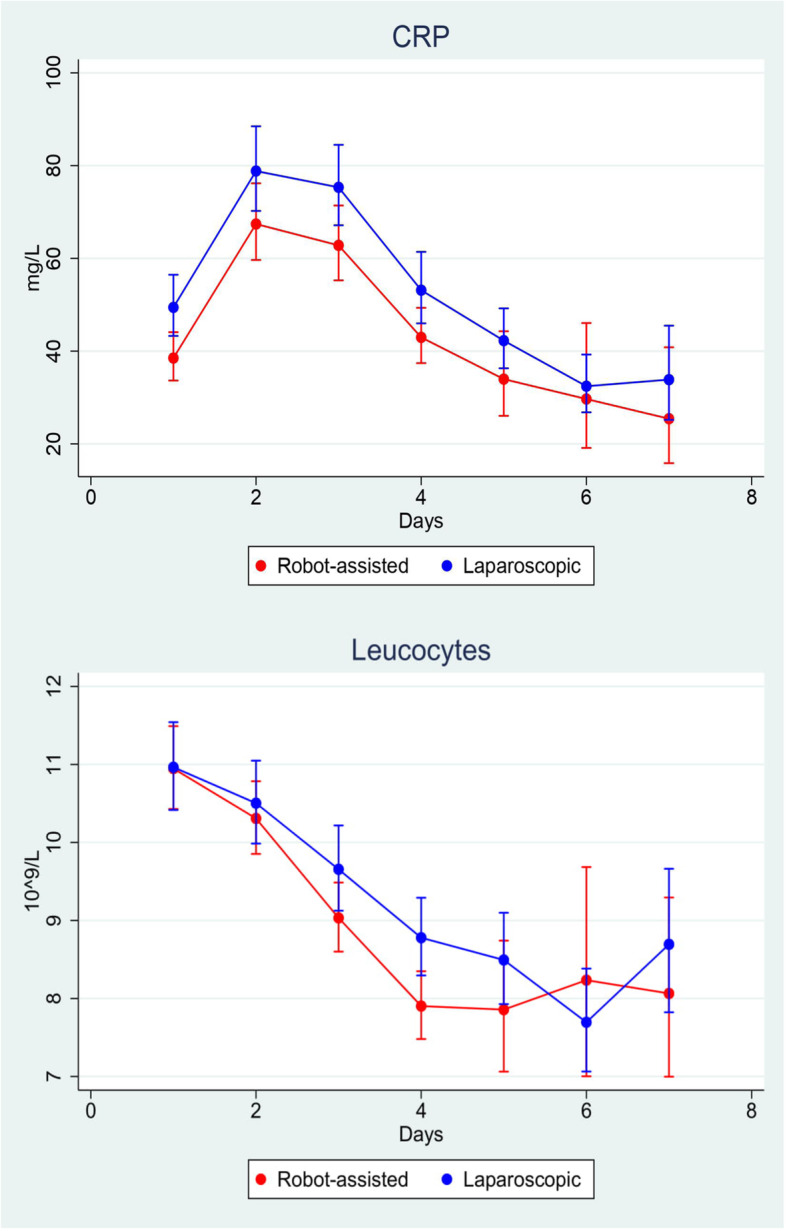
Distribution of postoperative C-reactive protein (CRP) level and leucocyte count day 1–7 in RCS versus LCS

The time of hospitalization was reduced by a median of 1 day in the RCS group compared to the LCS group (4 days (4, 5) versus 5 days (4–7), p < 0.001). Time to first stool favored LCS (2 days (1–3) versus RCS, 3 days (2, 3), p = 0.033). No differences in postoperative surgical and medical complication rates were demonstrated between the two groups (Table 3). CRP concentration was significantly lower in the RCS group on day 1, 3, and 4 postoperatively. Similarly, the leukocyte concentration was significantly lower in the RCS group on postoperative day 4 but otherwise did not differ significantly in the postoperative period (Fig. 2, Table 3).

**Table 3:** Comparison of postoperative outcomes and complication rates of included patients operated for malignant colorectal neoplasm. *RCS* robot-assisted colorectal surgery, *LCS* laparoscopic colorectal surgery, *CRP* C-reactive protein

Postoperative outcomes and complication rates	Level	RCS, n =143	LCS, n = 155	p-value
Length of hospital stay, median (IQR), days		4 (4–5)	5 (4–7)	< 0.001^b^
Time to first flatus, median (IQR), days		2 (1–2)	1 (1–2)	0.145^b^
Time to first stool, median (IQR), days		3 (2–3)	2 (1–3)	0.033^b^
Surgical complication rate (Clavien Dindo), n (%)	I-II	7 (4.9%)	11 (7.1%)	0.519^a^
	III-V	8 (5.6%)	13 (8.4%)	
Type of surgical complication rate (Clavien Dindo IV), n (%)	Postoperative bleeding	3 (2.1%)	4 (2.6%)	
	Ileus	1 (0.7%)	2 (1.3%)	
	Wound abscess	3 (2.1%)	3 (1.9%)	
	Intraabdominal abscess	0 (0%)	1 (0.6%)	
	Stoma complication rate	1 (0.7%)	5 (3.2%)	
	Anastomotic leakage	0 (0%)	1 (0.6%)	
	Other	7 (4.9%)	8 (5.2%)	
Medical complication rate (Clavien Dindo), n (%)	I-II	18 (12.5%)	28 (18.1%)	0.340^a^
	III-V	6 (4.2%)	3 (1.9%)	
Type of medical complication rate (Clavien Dindo I-V), n (%)	Acute myocardial infarction	0 (0%)	1 (0.6%)	
	Pneumonia	3 (2.1%)	8 (5.2%)	
	Cardiac insufficiency	3 (2.1%)	3 (1.9%)	
	Pulmonary embolism	1 (0.7%)	3 (1.9%)	
	Respiratory insufficiency	3 (2.1%)	5 (3.2%)	
	Renal insufficiency	2 (1.4%)	4 (2.6%)	
	Sepsis	1 (0.7%)	0 (0%)	
	Other	11 (7.7%)	7 (4.5%)	
Mortality, n (%)		3 (2.1%)	1 (0.6%)	0.500^c^
CRP, median (IQR), mg/L	Day 1	43 (26–61)	50 (31–81)	0.027^b^
	Day 2	70.5 (48–120)	82 (46–127)	0.133^b^
	Day 3	64 (40–105)	82 (46–127)	0.037^b^
	Day 4	43 (26–72.5)	53 (34–93)	0.017^b^
	Day 5	30 (19–66)	44 (26–67)	0.169^b^
	Day 6	24 (14–78)	32 (20–62)	0.470^b^
	Day 7	27 (18–54)	35.5 (21–64)	0.358^b^
Leukocytes, median (IQR), 10^9^/L	Day 1	11 (9–13)	11 (9–13)	0.781^b^
	Day 2	11 (9–12)	10 (8–12)	0.928^b^
	Day 3	9 (7–11)	10 (8–12)	0.158^b^
	Day 4	8 (6–10)	8 (7–11)	0.016^b^
	Day 5	8 (6–11)	9 (7–10)	0.169^b^
	Day 6	8 (7–10)	8 (6–10)	0.557^b^
	Day 7	8.4 (±2.3)	9.0 (±2.9)	0.409^b^

^a^*χ*^2^-test

^b^Wilcoxon rank sum test

### Pathologic outcome

No differences were observed in T-, N-stage or amount of harvested lymph nodes between the patients operated with RCS or LCS (Table 4). A significant number of patients with distant disease occurred in the LCS group (n = 3 (1.9%) compared to the RCS group (n = 0), p = 0.043).

**Table 4:** Histopathological characteristics in patients operated for colorectal malignant neoplasm by RCS and LCS surgery. *T-stage* size of primary tumor, *N-stage* degree of lymph node dissemination, *M-stage* locoregional lymph node or distant spread, *RCS* robot-assisted colorectal surgery, *LCS* laparoscopic colorectal surgery

Oncologic outcomes	Level	RCS (n = 143)	LCS (n = 155)	p-value
T-stage, n (%)	0	1 (0.7%)	5 (3.2%)	0.237^a^
	1	35 (24.5%)	27 (17.4%)	
	2	24 (16.8%)	28 (18.1%)	
	3	70 (49.0%)	74 (47.7%)	
	4	13 (9.1%)	21 (13.5%)	
N-stage, n (%)	0	89 (62.2%)	82 (52.9%)	0.087^a^
	1	31 (21.7%)	50 (32.3%)	
	2	23 (16.1%)	23 (14.8%)	
M-stage, n (%)	0	143 (100%)	152 (98.1%)	0.043^a^
	1	0 (0%)	3 (1.9%)	
Harvested lymph nodes, median (IQR)		37 (28–47)	34 (25–44)	0.0610^b^
Microradical resection, n (%)	R0	137 (95.8%)	145 (93.5%)	0.388^a^
	R1	6 (4.2%)	10 (6.5 %)	

^a^*χ*^2^-test

^b^Wilcoxon rank sum test

^c^Fisher’s exact test

### Multivariate regression analysis

A multivariate regression analysis was performed. The analysis was adjusted for T-stage, ASA-score, neoadjuvant chemotherapy, BMI, age, type of resection (colonic or rectal), and temporary diverting ileostomy formation. Intra- and postoperative surgical and medical complication rates, operative time, length of hospital stay, intraoperative blood loss, time to first flatus and stool, amount of harvested lymph nodes, CRP, and leukocyte count were analyzed (Table 5). The multivariate analysis demonstrated a statistically significant decrease in surgical time (IRR = 0.98, 95%CI = 0.97–0.99, p = 0.005), and a reduction in the amount of harvested lymph nodes (IRR = 0.91, 95%CI = 0.88–0.95, p < 0.001) in the LCS group. Time to first flatus was changed by this adjustment compared to the univariate analysis, and did not differ significantly between the two surgical methods (IRR=1.01, 95%CI = 0.84–1.21, p = 0.935). The remaining intra- and postoperative outcomes outlined in the univariate analysis were not affected by the regression analysis.

**Table 5:** Multiple regression analysis of intraoperative outcomes, postoperative complication rates, and biochemical markers of systemic inflammation in RCS and LCS surgery for colorectal cancer. Represented values of regression analysis for LCS where RCS is the reference value. All analyses were adjusted for T-stage, ASA-score, neoadjuvant chemotherapy, BMI, age, type of resection (colonic or rectal), and temporary diverting ileostomy formation. *OR* odds ratio, *CI* confidence interval, *IRR* incidence rate ratio, *EC* exponentiated coefficient

Variable	Coefficient		95% CI	P-value
	RCS (n = 143)	LCS (n = 155)		
Surgical complication rate (OR, 95%CI)	1	1.51	0.69–3.29	0.300^a^
Medical complication rate (OR, 95%CI)	1	0.97	0.51–1.87	0.941^a^
Intraoperative blood loss (IRR, 95%CI)	1	1.78	1.35–2.35	< 0.001^b^
Operative time (IRR, 95%CI)	1	0.98	0.97–0.99	0.005^c^
Length of stay (IRR, 95%CI)	1	1.12	1.02–1.24	0.018^c^
Time to first flatus (IRR, 95%CI)	1	1.01	0.84–1.21	0.933^c^
Time to first stool (IRR, 95%CI)	1	1.01	0.87–1.17	0.935^c^
Harvested lymph nodes (IRR, 95%CI)	1	0.91	0.88–0.95	< 0.001^c^
CRP (EC, 95%CI)	1	1.23	1.06–1.44	0.008^d^
Leukocytes (EC, 95%CI)	1	1.04	0.98–1.11	0.177^d^

^a^Ordinal logistic regression

^b^Negative binomial regression

^c^Poisson regression

^d^Multilevel mixed effect linear regression

## Discussion

The present study demonstrates a significant reduction in length of hospital stay, intraoperative blood loss, and inflammatory stress response measured by CRP in patients undergoing colorectal resection for cancer with RCS compared to the LCS. A multivariate regression analysis with adjustment for clinically relevant confounders demonstrated an additional statistically significant reduction in the operative time in LCS, and the amount of harvested lymph nodes favorized RCS. No difference was found in surgical or medical morbidity, time to first flatus or stool, conversion rate to open surgery, and postoperative leukocyte count between the two groups in multivariate analyses.

Comparison of RCS and LCS for malignant disease remains poorly investigated. In 2012, a prospective randomized controlled trial examined the length of stay as a primary outcome and reported no difference between the two surgery techniques [2].

Recent systematic reviews and meta-analyses comparing surgical efficacy and safety of RCS and LCS favor RCS concerning several intra- and postoperative outcomes. These outcomes include lower conversion rates, intraoperative blood loss, decreased overall morbidity, earlier hospital discharge, and earlier establishment of bowel function [8, 13, 14, 21–23]. In this study, there was a non-significant conversion rate of 1.4% in the RCS group and 3.9% in the LCS group, p = 0.187. Solaini et al. reported a significant conversion rate to open surgery in LCS resections (RR 1.7; 95% CI (1.1–2.6), p = 0.02). This finding has also been confirmed in other systematic reviews and meta-analyses [3, 8, 24–27]. Despite more patients in our population receiving combined chemo-/radiotherapy, no significant difference in conversion rates could be detected between the surgical groups. Patients were equally distributed in the RCS and LCS group regarding ASA-scores and BMI, which can be attributed as a risk factor for an increased conversion rate. Due to a longstanding experience with minimally invasive colorectal surgery in our institution, we have a high threshold regarding conversion to open surgery. In a recent randomized controlled trial by Jayne et al., no significant difference in the conversion rate between RCS and LCS was found in patients undergoing rectal cancer surgery [7]. The low conversion rates in the RCS group may be attributed to a predominance of colonic resection. Colonic resections can be technically less demanding to perform compared to rectal resections — especially if the surgery is preceded by neoadjuvant chemo-/radiotherapy. However, conversion rates in the LCS group for patients receiving neoadjuvant radiotherapy were higher, but non-significant. Factors that may complicate the surgical procedure and induce this higher conversion rate include radiotherapy to the pelvic floor causing fibrosis, edema, inflammation, and necrosis [28]. Neoadjuvant radiotherapy is associated with a higher risk of postoperative surgical complication rates and delayed perineal wound healing following abdominoperineal resection [29]. None of these complication rates were overrepresented in our study.

In contrast to other studies, operative time was no longer for RCS than LCS [4, 10, 24–26, 30]. In the multivariate analysis a statistically significant, not clinically relevant reduction in the operating time was demonstrated in LCS group (IRR = 0.98, 95% CI 0.97–0.99, p = 0.005). However, previous meta-analyses have been conducted on predominantly observational studies, whereby there is a risk of misinterpretation of total procedure times and surgical times [31–33]. The widely criticized fact by RCS is the setup and docking time of the robotic console. The prolonged surgical times cannot be attributed to simple factors but rather the joint effort of the limited number of certified RCS surgeons, dedicated operating nurses and/or anesthesiology team. Improved RCS technological development and a transition from DaVinci Si ® to the Xi ® model, and a dedicated robotic team can reduce total operation time. Several studies have confirmed a reduction in both docking and total operating times using the DaVinci Xi ® model, with an average of 21 cases needed to reach a statistically significant reduction in the docking time [34–36]. The technological development of RCS and the da Vinci Xi ® model’s introduction allows the surgeon more freedom and the ability to perform even technically demanding procedures that previously have been difficult to perform laparoscopically. The stable and precise high definition camera, which the surgeon independently can maneuver and the higher degree of free movement of robotic arm joints contributes to better hemostasis [37]. Although both surgical modalities are minimally invasive, RCS is presumed to be associated with a gentler manipulation with organs.

Postoperative CRP levels were significantly lower in RCS and can predict the inflammatory stress response induced by surgery. There is sparse literature reporting on the systemic inflammatory response in RCS compared to LCS [38]. Previous studies have mostly compared the systemic inflammatory response in RCS to open colorectal surgery. RCS was associated with a lower inflammatory stress response compared to open surgery [39, 40]. A prospective, non-randomized study comparing RCS (n = 30) and LCS surgery (n = 120) for early gastric adenocarcinoma reported a lower postoperative CRP and interleukin-6 response in the LCS group. In this study, there was an unequal distribution of patients, lack of randomization, and usage of older da Vinci robotic ® technology. These factors may have contributed to a lower postoperative inflammatory response in the LCS group. The perioperative stress response initiated by oncological surgery is a complex interplay of inflammatory and metabolic reactions [41, 42]. Previous studies found a correlation between the degree of local and systemic inflammation generated by the surgical trauma, risk of local and distant recurrence thereby increasing morbidity and mortality rates [41–43]. Minimally invasive surgery has replaced open surgery due to improved clinical short-term and long-term oncological outcomes [2, 44, 45]. The use of more gentle surgical techniques that minimize intraoperative bleeding, amounts of blood transfusions, and reduce manipulation of the primary tumor will contribute to less surgical trauma, and reduce the risk of escaped circulating tumor cells compared to open surgery [43]. In addition to Shibata et al., the perioperative stress response caused by RCS versus LCS has been sparsely studied in the literature [46]. This was a prospective, non-RCT and included a small, unevenly distributed cohort (n = 46) of both RCS, LCS, and open surgery for rectal cancer. Besides a higher stress response in the open surgery group, no difference was demonstrated between RCS and LCS.

Regarding oncological outcomes, the microradical resection rate was not statistically different between the groups. Our study showed a significantly lower amount of harvested lymph nodes in LCS group from the multivariate analysis. Many existing reports examine the rate of harvested lymph, and the majority report no differences between the two operation methods [2, 10, 25–27, 47, 48]. However, a large Danish register-based observational study including a total of 8104 LCS and 511 RCS procedures for colorectal cancer showed the risk of achieving a microradical resection in colon cancer was significantly higher using LCS, and higher but non-significant for rectal cancer in patients undergoing RCS [49].

The most important limitation of this study is the retrospective design. Patients were preoperatively balanced between the two surgical procedure groups regarding age distribution, BMI, and ASA-score. There was a selection of patients who had received neoadjuvant oncological treatment in favor of LCS. Due to an uneven distribution of patients having a temporary diverting loop ileostomy after rectal resection with an overrepresentation in the LCS group, an adjustment was performed in the multivariate regression analysis. These patients did not have a significantly increased rate of conversion or postoperative morbidity in either of the surgical groups. To minimize the risk of selection bias, we performed a multivariate regression analysis adjusting for clinically relevant confounders. Apart from time to first stool, none of the univariate analyses were non-significant by these adjustments.

The future possibilities of the rapidly evolving robotic technology result from improved software, telerobotics, ergonomics, and elimination of technical deficiencies. These advantages indicate that robot-assisted surgery may be superior to laparoscopic surgery in certain groups of patients with colorectal cancer. If improved inflammatory postoperative stress response can be demonstrated in RCS versus LCS in prospective clinical studies, robotic technology may improve long-term survival due to a more minimized tissue traumatization.

## Conclusion

RCS is a well-established surgical method. This study demonstrated the superiority of RCS compared to LCS in regard to surgical safety and efficacy. Lower intraoperative blood loss, shortened hospital stay, and a lower postoperative inflammatory stress response were observed in the RCS group. There is a need to evaluate the potential benefits of RCS, focusing on differences in inflammation caused by the two surgical methods in randomized and prospective studies.

## Data Availability

Data can be required from the corresponding author.

## Abbreviations

LCS: Laparoscopic colorectal surgery
RCS: Robot-assisted colorectal surgery
BMI: Body mass index
ASA: American Society of Anesthesiologists
TNM: TNM classification of malignant tumors
CRP: C-reactive protein
CT: Computer tomography
CME: Central mesocolic excision
OR: Odds ratio
CI: Confidence interval
IRR: Incidence rate ratio
EC: Exponentiated coefficient
